# Methodological considerations in the combined methylphenidate and physical activity trial for cancer-related fatigue in immunotherapy patients

**DOI:** 10.1017/S1478951526102661

**Published:** 2026-05-14

**Authors:** Dwi Sona, Titin Indah Pratiwi, Wiryo Nuryono, Mochamad Nursalim

**Affiliations:** 1 Department of Education, Universitas Negeri Surabaya, Surabaya, Indonesia; 2 Department of Education, Mulawarman University Faculty of Teacher Training and Education, Samarinda, Indonesia

Cancer-related fatigue (CRF) remains one of the most debilitating and undertreated symptoms in patients with advanced cancer-receiving immunotherapy, affecting up to 40% of those on anti-PD-1 agents and substantially impairing quality of life and treatment adherence (Kiss et al. 2022). We read with great interest the pilot randomized controlled trial by Yennurajalingam et al. (2026), which explored the effects of combining methylphenidate (MP) with a standardized physical activity (PA) intervention on CRF in patients with advanced cancer receiving anti-PD-1 immunotherapy. The authors are to be commended for addressing an important clinical gap with a mechanistically sound rationale: while MP targets central nervous system arousal via dopamine and norepinephrine reuptake inhibition, PA exerts immunomodulatory effects through reductions in pro-inflammatory cytokines implicated in CRF. Nevertheless, we wish to offer several methodological comments that we believe are important for the interpretation and future replication of these findings.

First, the study was substantially underpowered, enrolling only 40 of the planned 70 participants (57% of target), with only 34 evaluable patients ultimately analyzed. This shortfall was largely attributed to disruptions caused by the COVID-19 pandemic. Compounding this limitation, the authors chose a 2:1 randomization ratio, resulting in only 11 evaluable patients in the placebo arm. While the authors acknowledge that this design was intended to enrich the intervention arm for within-group analysis, such a configuration renders any between-group comparison statistically meaningless. This is particularly concerning given that both arms demonstrated clinically significant improvements in FACIT-F scores – with effect sizes of 0.87 in the MP + PA arm and 0.74 in the Pl + PA arm – yet no formal between-group statistical test was reported to substantiate claims of differential efficacy. Future adequately powered trials must incorporate conventional 1:1 randomization with sufficient sample sizes to detect between-group differences, especially given the magnitude of placebo response observed (Stone et al. 2024).

Second, the integrity of the physical activity intervention warrants scrutiny. Due to pandemic-related constraints, objective assessment of PA adherence was not systematically conducted. No wearable accelerometers, pedometers, or verified activity logs were employed to confirm that participants actually performed the prescribed resistance and walking exercises. This represents a critical confound: if PA adherence varied substantially between individuals or arms, the observed fatigue improvements cannot be confidently attributed to the combined intervention. Furthermore, the 2-week intervention duration is considerably shorter than the 4–12 week periods recommended by major guidelines for PA interventions in cancer populations (Ligibel et al. 2022). Such a brief window may only capture acute symptomatic relief rather than sustained physiological adaptation, limiting the clinical relevance of the findings.

Third, the statistical approach raises concerns regarding type I error inflation. The authors tested a large number of secondary outcomes – spanning PROMIS-F, 5 MFSI-SF subscales, 4 FACT-G domains, HADS, and ESAS – without applying any correction for multiple comparisons such as Bonferroni adjustment or false discovery rate correction. In a pilot study of this size, this substantially increases the risk of false-positive findings. Additionally, the absence of a formal blinding integrity assessment is a notable omission. Methylphenidate produces recognizable side effects such as dry mouth, palpitations, and dizziness, as evidenced by the 21 adverse events recorded in the MP + PA arm versus only 1 in the placebo arm. This stark imbalance strongly suggests that unblinding may have occurred, potentially introducing performance and detection bias that could have inflated the reported fatigue improvements in the active treatment group (Centeno et al. 2022).

Fourth, the generalizability of these findings is considerably limited by the demographic homogeneity of the study sample, in which 95% of participants were White or Caucasian. Cancer patients from diverse ethnic backgrounds, including Asian, African American, and Latino populations, may experience CRF differently due to variations in immune activation patterns, psychosocial stressors, and pharmacogenomic responses to methylphenidate. Moreover, the inclusion of heterogeneous cancer diagnoses – ranging from melanoma (52.5%) to genitourinary, thoracic, and head and neck cancers – without subgroup analyses precludes understanding whether the intervention’s efficacy is uniform across tumor types. Future phase III trials should prioritize diverse recruitment and incorporate tumor-specific subgroup analyses to ensure that the benefits of MP + PA are equitably characterized across the population of patients receiving anti-PD-1 immunotherapy.

In conclusion, while Yennurajalingam et al. (2026) present an encouraging and mechanistically compelling pilot study supporting the combined use of methylphenidate and physical activity for CRF in immunotherapy-treated patients, the findings must be interpreted with caution given the underpowered sample, unverified PA adherence, risk of unblinding, multiple comparison errors, and limited generalizability. We hope these methodological observations contribute constructively to the design of future well-powered, rigorously controlled phase III trials that can more definitively establish the role of MP + PA in the multimodal management of cancer-related fatigue.
